# Ultrasonic Microfluidic Method Used for siHSP47 Loaded in Human Embryonic Kidney Cell-Derived Exosomes for Inhibiting TGF-β1 Induced Fibroblast Differentiation and Migration

**DOI:** 10.3390/ijms26010382

**Published:** 2025-01-04

**Authors:** Ranran Yuan, Zhen Mu, Houqian Zhang, Jianwei Guo, Yu Tian, Quanlin Xin, Xiaojing Zhu, Zhengya Dong, Hongbo Wang, Yanan Shi

**Affiliations:** 1 School of Pharmacy, Key Laboratory of Molecular Pharmacology and Drug Evaluation, Ministry of Education, Collaborative Innovation Center of Advanced Drug Delivery System and Biotech Drugs in Universities of Shandong, Yantai University, Yantai 264005, China; 15239913157@163.com (R.Y.); muz990122@163.com (Z.M.); zhanghouqian99@163.com (H.Z.); 18769933062@163.com (Y.T.); xinquanlin813@163.com (Q.X.); 2 Shandong Laboratory of Advanced Materials and Green Manufacturing at Yantai, Yantai 264006, China; jwguo@amgm.ac.cn; 3 Guangdong Laboratory of Chemistry and Fine Chemical Engineering, Shantou 515031, China; zhuxj@ccelab.com.cn (X.Z.); zydong@ccelab.com.cn (Z.D.)

**Keywords:** siRNA, HSP47, IPF, EXOs, ultrasonic microfluidic method

## Abstract

Idiopathic pulmonary fibrosis (IPF) is a chronic, progressive, and devastating lung disorder. In response to transforming growth factor-β (TGF-β), normal lung cells proliferate and differentiate into myofibroblasts, which are instrumental in promoting disease progression. Small interfering RNA (siRNA) targeting heat shock protein 47 (HSP47) has been demonstrated to alleviate IPF by blocking collagen synthesis and secretion. Exosomes (EXOs) have been investigated for drug delivery due to their superior carrier properties. However, their loading efficiency has been a limiting factor in widely application as drug carriers. In this study, an ultrasonic microfluidic method was employed to enhance the loading efficiency of siHSP47 into EXOs, achieving 31.1% efficiency rate. EXOs were isolated from human embryonic kidney cells (293F) and loaded with siHSP47 (EXO-siHSP47). The findings indicated that EXO-siHSP47 penetrated the collagen barrier and effectively silenced HSP47 expression in activated fibroblasts in vitro. Western blotting and immunofluorescence analyses confirmed that EXO-siHSP47 significantly reduced the secretion and deposition of extracellular matrix (ECM) proteins. Wound healing and Transwell migration assays demonstrated that EXO-siHSP47 inhibited fibroblast differentiation and migration. In conclusion, 293F-derived EXOs loaded with siHSP47 present a promising therapeutic strategy for IPF.

## 1. Introduction

Idiopathic pulmonary fibrosis (IPF) is the most prevalent among idiopathic interstitial lung disorders [1]. It is characterized by progressiveness, high mortality, and a dearth of effective therapeutic options. The aberrant activation of transforming growth factor-β1 (TGF-β1) during pulmonary fibrogenesis recruits myofibroblasts from multiple cellular origins [1]. Activated myofibroblasts contribute to the excessive accumulation of extracellular matrix (ECM) components. IPF is marked by excessive collagen production and accumulation, leading to the transformation of healthy tissue into fibrotic scar tissue. This process disrupts gas exchange between the alveoli and pulmonary vessels, ultimately causing breathlessness and a decline in lung function [2]. The complex pathogenesis of IPF complicates the development of targeted therapies [3]. Currently, only two drugs have received regulatory approval for the treatment of IPF: nintedanib and pirfenidone. Although these two drugs extend patient survival [4,5], the benefits are limited, with survival extending to 20 months for nintedanib and 19 months for pirfenidone [6], and the administration of both drugs is associated with mild or moderate gastrointestinal side effects due to off-target effects [7]. Lung transplantation remains the sole therapy demonstrated to markedly enhance survival. Nevertheless, the limited availability of suitable donor lungs and the substantial mortality risk associated with the procedure have led to relatively low transplantation success rates [8].

Heat shock protein 47 (HSP47), a collagen-specific molecular chaperone, located in the endoplasmic reticulum, is essential for the proper folding of procollagen. Typically, HSP47 expression aligns with collagen production across different cells and tissues, supporting blood circulation, hemostasis, and the preservation of tissue structural integrity [9]. In fibrotic cells, HSP47 is upregulated in response to TGF-β1 stimulation, alongside increased expression of collagen I (COL-1) and collagen III [10,11]. HSP47 has been investigated as a therapeutic target in various fibrotic diseases, including pulmonary fibrosis [9,12], liver fibrosis [13], skin fibrosis [14]. However, no specific inhibitor of HSP47 currently exists. Silencing HSP47 expression via RNA interference (RNAi) technology induces apoptosis in myofibroblasts [15], which subsequently reduces COL-1 and fibronectin (FN) expression [16].

Exosomes (EXOs) are small vesicles, ranging in size from 30 to 150 nm, that are released by cells. Their presence was initially identified in sheep reticulocytes through electron microscopy [17]. Subsequently, EXOs were discovered in various cell types, including stem cells [18,19,20], macrophages [21,22], fibroblasts [23,24], and tumor cells [25,26]. EXOs are formed through plasma membrane invagination, generating intraluminal vesicles which encapsulate cargo. These vesicles then fuse with the plasma membrane to release EXOs. EXOs can evade host immune activation or attenuate immunogenic responses due to their small size, biocompatibility and low immunogenicity [27]. The presence of CD47 on EXOs shields them from being engulfed by monocytes and macrophages, thereby promoting their persistence within the bloodstream [28]. Thus, EXOs are readily internalized and resistant to degradation by phagocytosis and lysosomes. EXOs have been widely employed as drug carriers for delivering siRNA in various diseases. Currently, several methods have been reported for loading siRNA into EXOs, including incubation, electroporation and sonication. While the incubation method typically exhibits low loading efficiency [29,30], its primary advantage is maintaining the structural integrity of both EXOs and siRNA. Electroporation is currently prevalent, achieving approximately 10–20% efficiency [31]. However, electroporation can cause particle enlargement or aggregation [32,33]. siRNA encapsulated EXOs using nano electroporation-based methods enable passive loading of cargo into EXOs within cells, effectively avoiding the structural damage to EXO membranes that can occur with conventional electroporation methods [34]. The sonication method also cause damage to EXOs, potentially affecting both the measurement of loading efficiency and the effectiveness of drug delivery. Sonication is considered gentler compared to electroporation. However, multiple ultrasound treatments are required to ensure adequate drug penetration [35]. This process may lead to a modest enlargement of the EXO particle size [20].

In this study, EXOs were isolated from human embryonic kidney (293F) cells (293F-EXOs). siHSP47 was then loaded into EXOs (EXO-siHSP47) using an ultrasonic microfluidic method for delivery to activated fibroblasts. This method utilizes the cavitation effect of the ultrasonic microreactor to facilitate the blending of 293F-EXOs with siHSP47, thereby achieving expeditious and uniform encapsulation. Compared to conventional sonication methods, the ultrasonic microfluidic method enables siRNA loading in less time with a single, low-power ultrasound pulse, minimizing damage to the membrane structure of EXOs and siRNA. In vitro evaluation of the antifibrotic effects of EXO-siHSP47 revealed a significant downregulation of ECM protein expression and a pronounced inhibition of fibroblast activation and migratory behaviors. Consequently, 293F-EXOs loaded with siHSP47 emerge as a promising candidate for the development of therapeutic strategies aimed at the amelioration of IPF.

## 2. Results

### 2.1. Isolation and Characterization of 293F-EXOs

**Figure 1:**
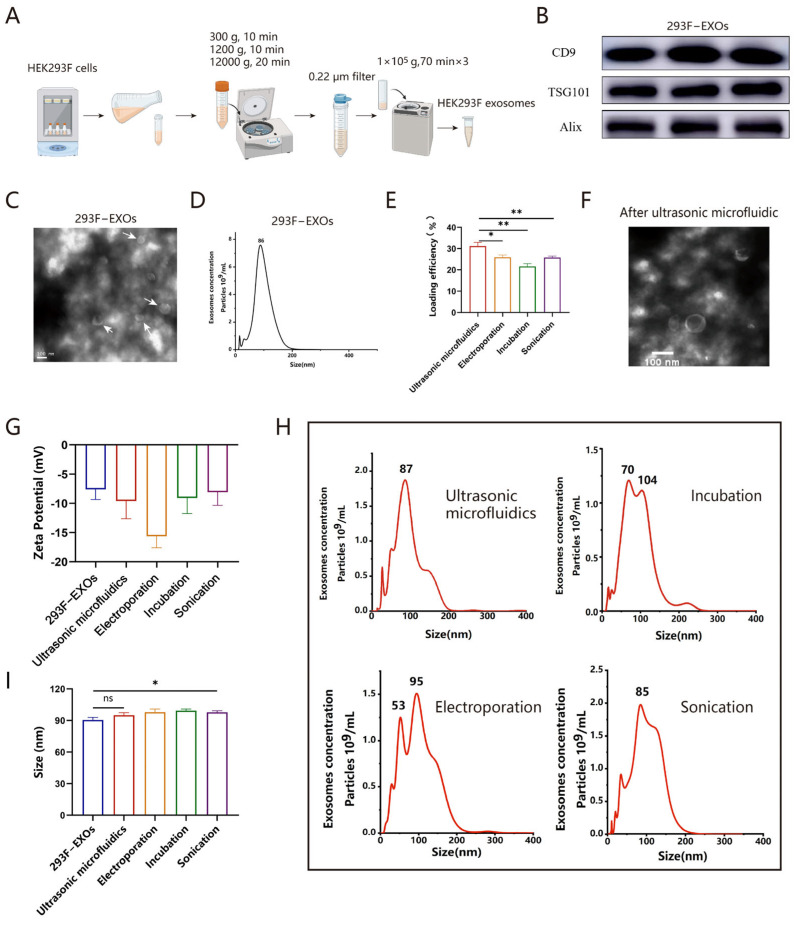
Characterization of 293F-EXOs and preparation of 293F-EXOs loaded with siRNA (EXO-siRNA). (**A**). The process of 293F-EXOs isolation. (**B**). Western blotting analysis of 293F-EXOs signature proteins (CD9, TSG101, and Alix). (**C**). Transmission electron microscopy (TEM) image of 293F-EXOs. (**D**). Nanoparticle tracking analysis (NTA) demonstrating the size distribution of 293F-EXOs. (**E**). The loading efficiency of various methods (ultrasonic microfluidics, electroporation, incubation, and sonication). (**F**). TEM image of EXO-siRNA obtained using ultrasonic microfluidic method. (**G**). The zeta potentials of EXO-siRNA were prepared by different methods. (**H**). NTA was used to analyze the particle sizes and size distributions of EXO-siRNA. (**I**). Changes in particle size (ns *p* > 0.05, * *p* < 0.05, ** *p* < 0.01).

The schematic diagram illustrating the process of obtaining 293F-EXOs is shown in Figure 1A. The 293F cells, cultured at a density of 5 × 10^6^ cells/mL, were subjected to differential centrifugation to collect the supernatant, followed by EXOs isolation. Transmission electron microscopy (TEM) results revealed a cup-shaped [36] with a double-layer membrane (Figure 1C), which is characteristic of EXOs. Nanoparticle tracking analysis (NTA) was conducted to detect the particle size and concentration of the EXOs, exhibiting a narrow peak in the size distribution, with an average particle size of approximately 90 nm, and the majority of particles measuring around 86 nm (Figure 1D). Specific EXO signature proteins, including CD9, TSG101, and Alix, were confirmed in the 293F-EXOs through Western blotting (Figure 1B), thereby validating the successful isolation of EXOs from 293F cells.

### 2.2. Preparation and Characterization of EXO-siRNA

293F-EXOs loaded with siRNA (EXO-siRNA) was prepared using ultrasonic microfluidics, electroporation, incubation, and sonication. Among these methods, ultrasonic microfluidics achieved the highest loading efficiency, reaching 31.1% (Figure 1E), while this method achieved a copy number of 6322.9 ± 816.2 copies/EXO (Supplementary Figure S1). The zeta potential of EXO-siRNA, as compared to that of unloaded EXOs, was significantly altered by the electroporation method (8 mV vs. 13 mV), while the zeta potential of EXO-siRNA prepared by ultrasonic microfluidics, incubation, and sonication remained largely unchanged (Figure 1G). TEM analysis of EXO-siRNA revealed no morphological changes in EXOs after ultrasonic microfluidic treatment (Figure 1F). The electroporation method increased particle size and resulted in the formation of siRNA aggregates, potentially due to the effects of the electroporation buffer [32,33]. The sonication method, although milder than electroporation, also marginally increased the particle size of EXOs. The ultrasonic microfluidic method has minimal impact on the particle size of EXOs (Figure 1H,I).

### 2.3. TGF-β1 Induced Fibroblasts to Transform into Myofibroblasts

**Figure 2:**
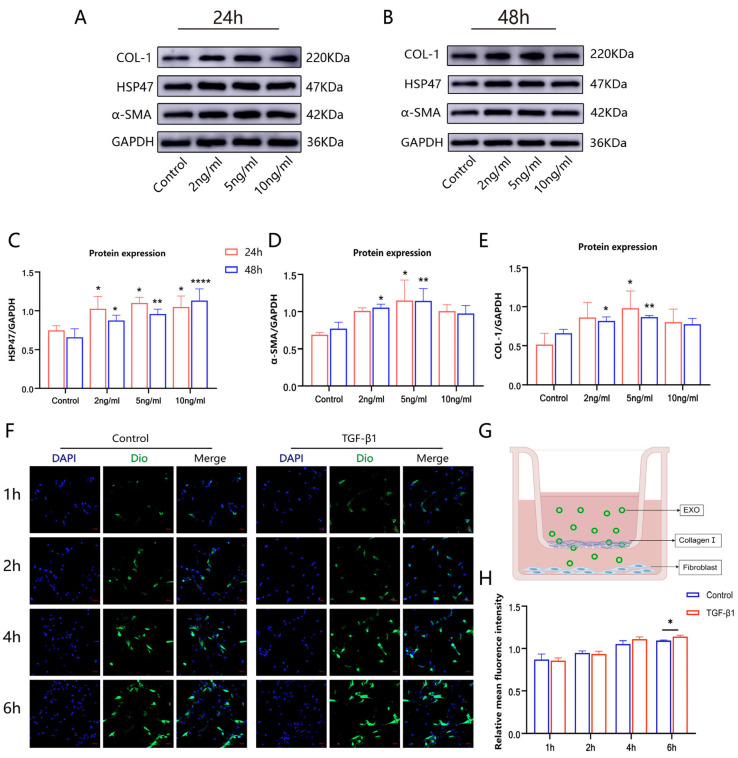
TGF-β1-induced concentration and collagen penetration. (**A**,**B**). Western blotting was used to analyze the expression of α-SMA, HSP47, and COL-1 in human fetal lung fibroblast 1 (HFL-1) cells treated with different concentrations of TGF-β1 (2 ng/mL, 5 ng/mL, and 10 ng/mL) for 24 and 48 h. (**C**–**E**). Relative expression levels of HSP47, α-SMA, and COL-1 were quantified. (**F**). Laser confocal microscopy images showing cell penetration through the collagen barrier into fibroblasts at 1, 2, 4, and 6 h. (**G**). Schematic diagram illustrating EXO penetration through collagen using a Transwell migration assay. (**H**). Fluorescence intensity analysis of cellular uptake of DiO-labeled EXOs at different time points. (* *p* < 0.05, ** *p* < 0.01, **** *p* < 0.0001).

TGF-β serves as a pivotal mediator in fibrogenesis, playing a crucial role in this process. It promotes the phenotypic conversion of fibroblasts into myofibroblasts [37]. Smooth muscle actin (α-SMA) is commonly used as a marker for myofibroblasts [38], serving as an indicator of myofibroblast activity. Fibroblasts were treated with cell culture medium containing 2, 5, and 10 ng/mL concentrations of TGF-β1 for 24 and 48 h, after which the expression of related proteins was analyzed by Western blotting (Figure 2A,B). Under TGF-β1 induction, the expression of HSP47 was upregulated (Figure 2C). At a concentration of 5 ng/mL, the expression of α-SMA and COL-1 increased (Figure 2D,E), indicating that the number of myofibroblasts had increased and fibroblasts had successfully transformed into myofibroblasts [39].

### 2.4. EXOs Penetrated the Collagen Barrier into Fibroblasts

ECM is a principal constituent of pulmonary fibrogenesis, with COL-1 produced by myofibroblasts [40], being its primary constituent. To evaluate the ability of EXOs to penetrate the collagen barrier, a Transwell polycarbonate membrane coated with COL-1 was used to simulate this barrier (Figure 2G). Laser confocal microscopy was employed to track EXOs uptake by cells at 1, 2, 4, and 6 h. The results showed that EXOs penetrated the collagen barrier and were taken up by fibroblasts in a time-dependent manner. EXOs were initially released by cells and readily internalized by other cells. Natural EXOs are known to target damaged tissues and cells [41], and physicochemical damage to receptor cells has been shown to enhance EXO uptake [42]. In this experiment, TGF-β1-induced cells treated with EXOs showed increased uptake by myofibroblasts, which are key effector cells responsible for ECM production in lung fibrosis [43]. This enhanced uptake may facilitate drug delivery to inflammatory cells, thereby inhibiting inflammation progression (Figure 2F,H).

### 2.5. siHSP47 Effectively Silence HSP47 Expression in Activated Fibroblasts

**Figure 3:**
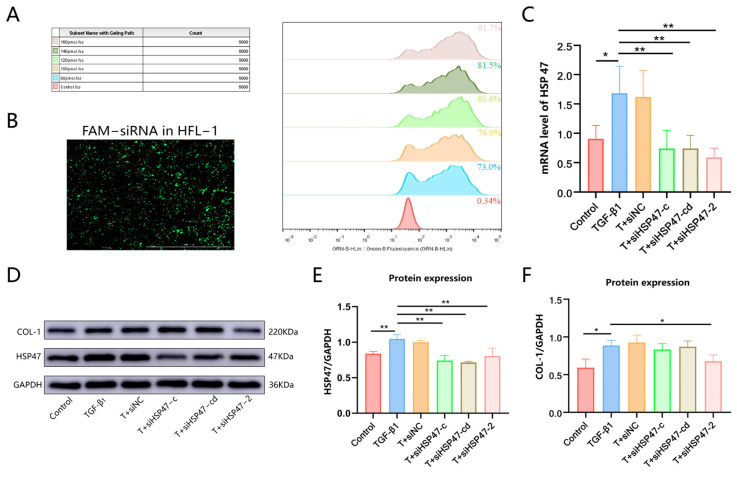
Transfection efficiency analysis and siHSP47 screening. (**A**). The flow cytometry image shows the transfection efficiency analysis of FAM-siRNA at different concentrations (0, 80, 100, 120, 140, and 160 nM). (**B**). Images of HFL-1 cells transfected with FAM-siRNA. (**C**). qRT-PCR results showing the expression of three siRNAs (siHSP47-c, siHSP47-cd, and siHSP47-2) in HFL-1 cells transfected at 120 nM for 24 h, assessing HSP47 mRNA levels. (**D**). Western blotting revealed the expression of HSP47 and COL-1 proteins in HFL-1 cells following transfection with three siRNAs at a concentration of 120 nM for 48 h. (**E**,**F**). Relative expression levels of HSP47 and COL-1 were quantified (* *p* < 0.05, ** *p* < 0.01).

HSP47 is a potent target for antifibrosis therapy, and previous studies have reported that pirfenidone can reduce HSP47 expression levels. However, there is currently no specific drug designed to target HSP47 [44]. Three siRNAs targeting HSP47 were synthesized, and transfection conditions were optimized using flow cytometry. It was found that cell transfection efficiency reached 80% when the siRNA concentration was 120 nM (Figure 3A,B). qRT-PCR and Western blotting results demonstrated that siHSP47-c, siHSP47-cd, and siHSP47-2 all reduced HSP47 mRNA (Figure 3C) and protein levels (Figure 3D,E). Among them, only siHSP47-2 significantly reduced COL-1 expression (Figure 3F). Subsequent experiments were conducted using siHSP47-2.

### 2.6. EXO-siHSP47 Inhibit TGF-β1 Induced Fibroblast Activation In Vitro

**Figure 4:**
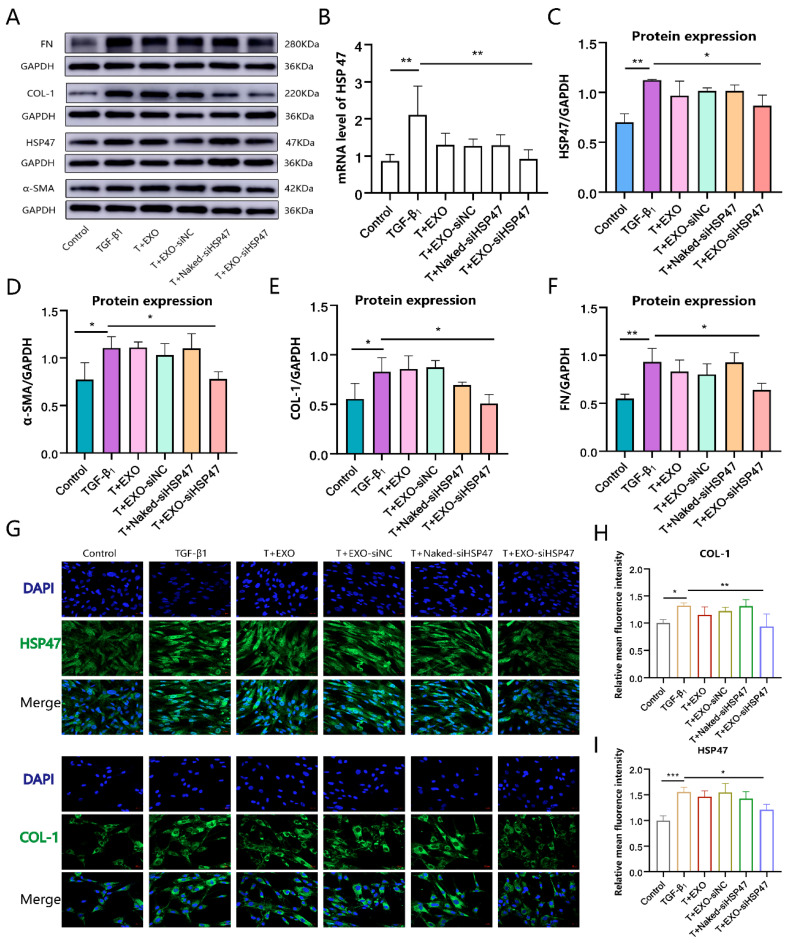
The EXO-siHSP47 reduces ECM deposition. (**A**). Western blotting was employed to analyze the expression levels of α-SMA, HSP47, COL-1, and FN. (**B**). HSP47 mRNA expression levels were assessed by qRT-PCR. (**C**–**F**). Relative expression levels of HSP47, α-SMA, COL-1, and FN were quantified. (**G**). Immunofluorescence staining was used to examine the expression levels of HSP47 and COL-1. (**H**,**I**). Fluorescence intensity plots for the quantification of HSP47 and COL-1 protein expression. The results are presented as the mean ± standard deviation (n = 3). (* *p* < 0.05, ** *p* < 0.01, *** *p* < 0.001).

The therapeutic potential of EXO-siHSP47 was assessed by applying it to fibroblasts stimulated with TGF-β1 for a duration of 24 h. Total RNA was extracted and evaluated through qRT-PCR, and the results demonstrated that EXO-siHSP47 efficiently silenced HSP47 mRNA expression (Figure 4B). After 48 h, the levels of HSP47 and ECM-associated proteins were evaluated using Western blotting (Figure 4A). The results demonstrated that the EXO-siHSP47-treated group exhibited reduced expression of HSP47 (Figure 4C), COL-1 (Figure 4E), and FN (Figure 4F), leading to a reduction in ECM deposition. Additionally, α-SMA expression was reduced (Figure 4D), indicating that EXO-siHSP47 effectively suppressed fibroblast activation. The immunofluorescence results further supported these findings (Figure 4G), showing reduced fluorescence intensity of HSP47 and COL-1 in the EXO-siHSP47-treated group (Figure 4H,I).

### 2.7. EXO-siHSP47 Inhibits TGF-β1 Induced Fibroblast Migration

**Figure 5:**
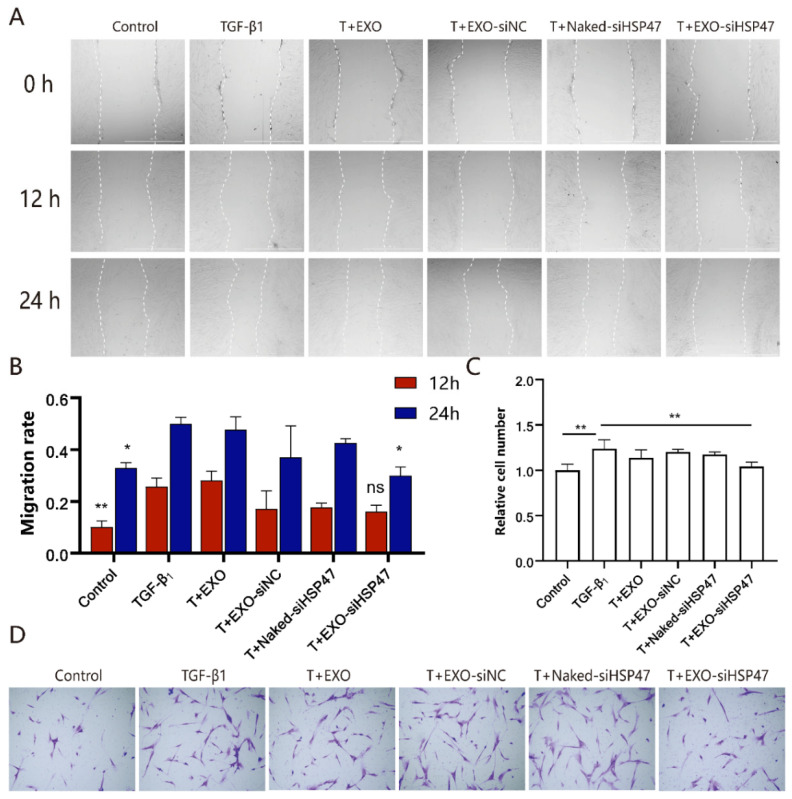
EXO-siHSP47 inhibit fibroblast activation and migration. (**A**). Migration of HFL-1 cells at different time points. (**B**). Analysis of the cell migration rate at 12 and 24 h. (**C**). Data analysis of the relative number of migrated cells. (**D**). Transwell migration assay, with purple indicating crystal violet-stained cell nuclei (ns *p* > 0.05, * *p* < 0.05, ** *p* < 0.01).

The inhibitory effect of siHSP47 on cell migration was evaluated using both wound healing and Transwell migration assays. The results demonstrated that EXO-siHSP47 significantly inhibited cell migration compared to the TGF-β1 group, whereas the EXO and naked siHSP47 groups showed no significant inhibitory effect (Figure 5A,B). The Transwell migration assay further revealed that the TGF-β1-induced inflammatory cell group displayed an increased number of cells crossing the membrane, whereas EXO-siHSP47 reduced the number of cells migrating through the polycarbonate membrane (Figure 5C,D), indicating that EXO-siHSP47 mitigated cellular inflammation by inhibiting cell migration.

## 3. Materials and Methods

### 3.1. Isolation of 293F-EXOs

A suspension of 293F cells (A14527, Thermo Fisher Scientific, Waltham, MA, USA) was dispensed into centrifuge tubes under sterile conditions. Using a high-speed benchtop centrifuge (Thermo Legend Micro21R, Thermo Fisher Scientific), the samples underwent sequential centrifugation at 300× *g*, 1200× *g* for 10 min, and 10,000× *g* for 20 min. The cell suspension was then filtered through a 0.22 μm membrane filter. The ultracentrifuge (Sorvall WX100+, Thermo Fisher Scientific, USA) was programmed to 4 °C and 100,000× *g* for 70 min. After centrifugation, 200 μL of PBS was added to the ultracentrifuge tube, and the precipitate was resuspended using a pipette. 293F-EXOs were stored at −80 °C.

### 3.2. Characterization of 293F-EXOs

TEM (JEM-1230; JEOL, Tokyo, Japan) was used to obtain images of 293F-derived EXOs. A 4 μL aliquot of the sample solution was applied to a copper grid and allowed to stand for 1 min. Next, 4 μL of phosphotungstic acid solution was placed for staining, applied for staining and left for 45 s. The sample was air-dried for observation. NTA was conducted using a nanoparticle tracking analyzer (Malvern Panalytical, Malvern, Worcestershire, UK) to evaluate the average size and particle concentration of EXOs in samples diluted with PBS. Zeta potential measurements were performed on approximately 1.5 mL of resuspended EXOs and EXO-siRNA introduced into the sample cells. Dynamic light scattering (DLS, Nanobrook 90PlusPALS, Brookhaven Instruments Corporation, Holtsville, NY, USA) was utilized to measure the zeta potential. Finally, EXOs were identified by Western blotting using signature proteins CD9, TSG101, and Alix.

### 3.3. Western Blotting Analysis

Total protein was extracted using RIPA buffer (MA0151, Meilunbio, Dalian, China) supplemented with protease inhibitors. Equal amounts of protein (8 μg) were resolved on 10% SDS-PAGE gels and subsequently transferred onto polyvinylidene fluoride (PVDF) membranes (Merck Millipore, Darmstadt, Hesse, Germany) via a protein transfer system (Bio-Rad, Hercules, CA, USA). PVDF membranes were blocked with NcmBlot blocking buffer (P30500, NCM Biotech, Seoul, South Korea) for 15 min and incubated overnight with primary antibodies specific for CD9 (ab275377, Abcam, Cambridge, MA, USA), TSG101 (Ab125011, Abcam), Alix (ab275377, Abcam), GAPDH (60004-1-Ig, Proteintech, Rosemont, IL, USA), HSP47 (A11698, ABclonal, Wuhan, China), α-SMA (A17910, ABclonal, China), COL-1 (72026, Cell Signaling Technology, Danvers, MA, USA), and FN (ab2413, Abcam). Secondary antibodies, goat anti-mouse (A0216, Beyotime, Nantong, China) and anti-rabbit (ab6721, Abcam) were used to incubate the PVDF membranes. Bands were visualized using chemiluminescence detection (Analytik, Jena, Germany) and analyzed with ImageJ software (ImageJ 1.54D).

### 3.4. Preparation of 293F-EXOs Loaded with siRNA

Ultrasonic microfluidic method: The siRNA was loaded into EXOs using an ultrasonic microreactor. The ultrasonic microreactor comprised an ultrasonic generator (TCR0120, Clangsonic Co., Ltd., Changzhou, China), an automatic syringe pump, and a microchannel [45]. In brief, the mixture of EXOs and siRNA was injected into the microchannel via the automatic syringe pump, with the inner diameter of the microchannel being 1.0 mm. The microchannel was coupled with the ultrasonic generator, which operated at a frequency of 20 kHz and was set to a power output of 30 W. The automatic syringe pump was configured to deliver the mixed solution into the microchannel at a flow rate of 200 μL/minute. Under the excitation of stable ultrasound waves delivered by the generator, transient pores formed in the EXOs, enabling rapid loading of siRNA into the EXOs.

Incubation method: siRNA and EXOs were mixed and incubated at room temperature for 3 h, with intermittent shaking every 30 min.

Electroporation method: EXOs and siRNA were combined at a defined ratio, and the resulting mixture was placed into an electroporation cuvette. Electroporation was conducted using the Gene Pulser Xcell™ system (Bio-Rad) with the following parameters: 160 V, and three pulses, each lasting 10.0 ms.

Sonication method: EXOs and siRNA were mixed at a specific ratio and placed in a water bath sonication chamber (BILON10-300B, Bilon Instrument Manufacturing Co., Ltd., Shenzhen, China). Sonication was performed at 100 W for 30 s, followed by 2 min on ice. This cycle was repeated 4 times, and the mixture was then incubated at room temperature for 1 h to restore the membrane structure.

Two portions of the treated mixture were collected. One portion was emulsified by adding 2% Triton X-100 (ST795, Beyotime). The total siRNA was quantified using the Quant-iT RiboGreen RNA kit (R11490, Invitrogen, Waltham, MA, USA). PBS containing EXOs was used as a control to measure the siRNA concentration. The loading efficiency was calculated using the following formula:

$$loading\;efficiency(\%)=\frac{w_{total}siRNA-w_{disassociate}siRNA}{w_{total}siRNA}\times 100\%$$

### 3.5. Cell Culture and Transfection

Human fetal lung fibroblast 1 (HFL-1) cells, provided by the Chinese Academy of Sciences (Shanghai, China), were cultured in Ham’s F-12K medium supplemented with 10% FBS (C0235, Gibco, Waltham, MA, USA), 1% non-essential amino acids, 1% GlutaMAX, and 1% sodium pyruvate, at 37 °C and 5% CO_2_. 293F cells were cultured in a shaker incubator (ZCZY-AS8E, Shanghai Zhichu Instruments Co., Ltd., Shanghai, China), at 37 °C, 90% relative humidity, and 5% CO_2_, using chemically defined, serum-free, protein-free Expi293™ Expression Culture (12338018, Thermo Fisher Scientific).

FAM-siRNA was transiently transfected into cells using a transfection reagent (GenePharma, Shanghai, China) at various concentrations according to the manufacturer’s instructions. After a 15 min resting period, the transfection reagent and siRNA were added to a medium containing 5% FBS. After 12 h of incubation, cells were digested and washed three times with PBS. The proportion of fluorescent cells was then detected using a flow cytometer (Guava^®^ easyCyte™ HT, Luminex, Shanghai, China) to determine the optimal transfection efficiency.

### 3.6. DiO-Labeled EXOs Penetration Assay Using Collagen Model In Vitro

Following the manufacturer’s protocol, 10 mg of DiO dye (C1038, Beyotime) was dissolved in DMSO to create a 5 mM stock solution, which was subsequently diluted with PBS to achieve a final concentration of 20 µM. The DiO dye and EXOs were mixed gently using sonication and incubated at 37 °C for 30 min. Excess dye was removed through ultrafiltration.

HFL-1 cells were plated into a Transwell chamber (Corning Incorporated, Corning, NY, USA) at a concentration of 4 × 10⁴ cells/mL and allowed to adhere. To simulate a collagen barrier, the polycarbonate membranes of the Transwell inserts were evenly coated with 50 µL of COL-1 (1 mg/mL) overnight. A 50 µL suspension containing DiO-labeled EXOs was added to the upper chamber for treatment. EXOs, at a concentration of 5 × 10^9^ particles per well, were co-cultured with the cells for 1, 2, 4, and 6 h. The cells were rinsed three times with PBS and fixed with 500 μL of 4% paraformaldehyde (PFA) for 15 min per well. Finally, the nuclei were stained using antifade mounting medium containing DAPI (P0131, Beyotime) and examined under a laser scanning confocal microscope (LSM800, Carl Zeiss AG, Oberkochen, Germany).

### 3.7. Quantitative Real-Time Polymerase Chain Reaction

Trizol reagent was utilized to isolate total RNA from the cell samples. Primer sequences were optimized by Sangon Biotech (Shanghai, China). The GAPDH primer sequences were forward *5′-GTCTCCTCTGACTTCAACAGCG-3′* and reverse *5′-ACCACCCTGTTGCTGTAGCCAA-3′*. The HSP47 primer sequences were forward *5′-AACCGTGGCTTCATGGTGACTC-3′* and reverse *5′-TGATGAGGCTGGAGAGCTTGTG-3′*. Reverse transcription was carried out using the Evo M-MLV Reverse Transcription Kit (Accurate Biotechnology Co., Ltd., Changsha, China). Quantitative Real-Time Polymerase Chain Reaction (qRT-PCR) was performed using the Cham Q SYBR Color qPCR Master Mix (Q421-03, Vazyme Biotech, Nanjing, China) on a CFX Connect real-time PCR detection system (Bio-Rad).

### 3.8. Immunofluorescence Staining and Microscopy

Cells treated with TGF-β1 or EXO-siHSP47 were cultured for 48 h, followed by two PBS washes and fixation with 4% PFA for 15 min. The cells were permeabilized for 10 min with 0.2% Triton X-100 and then blocked at room temperature for 15 min using QuickBlock™ Blocking Buffer for Immunol Staining (P0260, Beyotime). Afterward, the cells were incubated overnight at 4 °C with HSP47 and COL-1 antibodies at a 1:200 dilution. The following day, the cells were treated with a secondary antibody (A0423, Beyotime) for 1 h, and the nuclei were stained with DAPI. Finally, the cells were examined with laser confocal microscopy and the images were analyzed using ImageJ software.

### 3.9. Wound Healing Assay

HFL-1 cells were plated in 12-well plates at a concentration of 1 × 10^5^ cells/mL and grown until reaching 70% confluence. A sterile pipette tip was employed to create a scratch in the cell monolayer, followed by two PBS washes to eliminate any dislodged cells. The culture medium was then replaced with fresh medium containing TGF-β1 (5 ng/mL) along with various treatments. Cell migration was monitored at 0, 12, and 24 h using a live-cell imaging system (Cytation, BioTek™, Winooski, VT, USA), and the migration area was measured using ImageJ software.

### 3.10. Transwell Migration Assay

A Transwell chamber was utilized to evaluate the migratory capacity of HFL-1 cells. In brief, HFL-1 cells were harvested after 24 h for subsequent experiments. Approximately 4 × 10^4^ cells were resuspended in 200 μL of serum-free medium and seeded into the upper chamber. Meanwhile, 500 μL of medium containing 20% FBS, TGF-β1 (5 ng/mL), and various treatments (EXO, EXO-siNC, naked siHSP47, and EXO-siHSP47) were introduced into the lower chamber. After 48 h of incubation, cells that migrated to the lower chamber were stained with 0.1% crystal violet (G1063, Solarbio, Beijing, China) and quantified using a microscope (OLYMPUS BX53M, Olympus Corporation, Tokyo, Japan).

### 3.11. Statistical Analysis

The data are presented as the mean ± standard deviation. To assess the significance of differences among groups, two-tailed Student’s *t*-tests and one-way analysis of variance (ANOVA) were applied.

## 4. Discussion

In the progression of IPF, one of the primary causes is the overactivation of myofibroblasts, leading to excessive deposition of ECM. An efficacious therapeutic strategy to combat IPF progression is to prevent myofibroblast activation or diminish their population. EXOs have been utilized as drug carriers due to their excellent cell-homing properties and low toxicity. EXOs have demonstrated promising outcomes in delivering small nucleic acids, peptides, and proteins to lung cells to treat inflammation [46,47]. siRNAs are highly specific and potent in gene silencing. However, they are susceptible to nuclease degradation, which diminishes their cellular uptake [48]. Consequently, the necessity for suitable carriers for effective delivery is imperative. In this study, 293F cell-derived EXOs were loaded with siHSP47 to evaluate their antifibrotic effects in vitro. 293F cells, known for their rapid proliferation, enable the production of a substantial quantity of EXOs in a concise timeframe. From 500 mL of medium, 0.5–1.5 × 10^11^ EXO particles can be obtained after ultracentrifugation. Ultrasonic microfluidics efficiently loaded siHSP47 into 293F-EXOs with minimal damage. EXO-siHSP47 was shown to inhibit fibroblast differentiation and cell migration.

EXOs derived from 293F cells were isolated using differential ultracentrifugation, a commonly used method for EXO isolation [49]. Differential centrifugation removes cells, cellular debris, and larger extracellular vesicles. EXOs were isolated through ultracentrifugation at 1 × 10^5^ g and rinsed with PBS to eliminate impurities, such as proteins.

siRNA-based therapeutics have been implemented in the treatment of various diseases, with several receiving approval from the U.S. Food and Drug Administration and numerous others undergoing clinical trials [50]. Several siRNA targets have been explored for the treatment of IPF. Mechanistically, these targets can be classified into three categories: inhibition of macrophage polarization [51,52,53], epithelial-mesenchymal transition (EMT) [54], and fibroblast-to-myofibroblast transformation (FMT) [55,56,57]. The DNA repair enzyme 8-oxoguanine DNA glycosylase-1 (OGG1) plays a key role in modulating inflammation and metabolic syndrome. OGG1 interacts with SMAD7 to promote fibroblast proliferation and differentiation, whereas OGG1 siRNA suppresses myofibroblast migration and decreases the expression of ECM proteins at the cellular level [55]. Interleukin-11 (IL-11), a pro-fibrotic cytokine, inhibits fibroblast differentiation and reduces ECM deposition by delivering IL-11 siRNA to lung fibrotic inflammatory cells, thereby inhibiting ERK and SMAD2 signaling [56]. HSP47 is a crucial therapeutic target expressed by activated fibroblasts [58]. The expression of HSP47 is markedly upregulated in activated fibroblasts, and its absence impairs the proper folding of procollagen into a triple helix. This leads to the accumulation of misfolded procollagen in the endoplasmic reticulum, delaying collagen secretion [10], resulting in thinner collagen fibers [59]. Additionally, excessive accumulation of procollagen induces endoplasmic reticulum stress, leading to the death of activated fibroblasts [60]. The use of vitamin A-conjugated lipid nanoparticles (LNPs) loaded with siHSP47 has progressed to clinical trials for the treatment of liver fibrosis. These LNPs contain retinol, which binds to retinol-binding proteins expressed on hepatic stellate cells, facilitating targeted uptake by these cells. This process disrupts collagen formation and promotes stellate cell apoptosis, reducing or reversing fibrosis [61,62]. Similarly, siHSP47 delivered via the lipid nanoparticle ND-L02-s0201 has been demonstrated to decrease myofibroblast counts and enhance lung function in a rat model of pulmonary fibrosis induced by bleomycin. Cell-derived EXOs possess cell-specific properties and a bilayer membrane structure rich in membrane proteins, making them highly amenable to modification [63]. EXOs exhibit low immunogenicity, high stability, and strong tissue penetration, while modified EXOs enhance their potential for targeted delivery to disease sites. Compared to LNPs, EXOs exhibit superior distribution and retention of mRNA and protein cargo in the bronchioles and parenchyma following aerosol administration [64]. As drug delivery carriers, EXOs show promising potential.

Various methods are being explored to load siRNA into EXOs. The incubation method, while gentle, suffers from low loading efficiency. Optimizing incubation time and temperature can improve loading efficiency. Simple incubation efficiency is influenced by the pH of the solution. Anjana Jeyaram et al. investigated the loading efficiency of the incubation method with different pH gradients, and the results showed that the amount of EXO-loaded miRNA increased as pH became more acidic, reaching a peak at pH 2.5 [65]. The electroporation method offers higher loading efficiency but can damage EXO membranes due to the strong electric shock, increasing particle size. Kasper Bendix Johnsen et al. used NTA and TEM methods to investigate the effect of electroporation on EXOs. Their results showed that electroporation induces aggregate formation and increases particle size. EXO diameters range from 75 to 100 nm before electroporation to 100–500 nm after. An alginate buffer system was found to protect EXOs, mitigating the impact of electroporation on particle size [32]. Electroporation also induces siRNA aggregate formation, though this can be mitigated by appropriate electroporation buffer systems [33]. Buffers like phosphate and OptiPrep, which contain phosphate and hydroxide anions, can contribute to aggregate formation. In contrast, using a citrate-based buffer (pH 4.4) for electroporation reduces aggregate formation [33]. Maintaining EXO membrane integrity and drug stability is crucial for efficient loading and therapeutic efficacy. Therefore, selecting the appropriate electroporation buffer is critical. The ideal buffer should fulfill the dual requirements of protecting EXOs and the drug while not interfering with the formation of transient pores during electroporation. Electrode material should also be considered, as it can release hydroxide ions that contribute to particle formation. Compared to electroporation, sonication is milder and can achieve similar loading efficiency. After optimization, sonication has emerged as one of the more promising methods for drug loading. Sonication has been widely used to load various drugs into EXOs, including proteins, siRNAs, and chemical compounds. Sonication typically requires multiple cycles for adequate drug loading. For example, milk-derived EXOs loaded with siRNA achieved up to 24% loading efficiency after six 30-s sonication cycles [66]. In this study, ultrasonic microfluidics were employed to load siRNA into EXOs, achieving a loading efficiency exceeding 30%, surpassing both electroporation and sonication. The ultrasonic microfluidic method boasts superior liquid mixing performance. The liquid mixture of EXOs and siRNA, upon entering a microchannel with an inner diameter of 1.0 mm, is subjected to uniform and stable ultrasound excitation from the ultrasonic generator [45]. This provides intense mixing of the fluid, allowing for sample collection after a single, short-duration ultrasonic treatment. In contrast, conventional sonication methods require multiple cycles of approximately 30s water bath or probe sonication treatments. Such prolonged and repetitive treatments can cause significant damage to both exosomes and the loaded drugs [35], leading to poor reproducibility.

The cargo composition of EXOs is contingent upon their cellular origin. EXOs secreted by mesenchymal stem cells (MSC-EXOs) contain similar materials as their host cells, primarily RNAs, with miRNAs accounting for approximately 44%, covering over 150 species [67]. These miRNAs are associated with TGF-β, pro-fibrotic, and proliferative and apoptotic pathways in cells [68]. MSC-EXOs have been explored as a treatment for IPF, with investigations into their mechanisms of action within macrophages and in vivo models revealing their impact on the immune and redox systems in murine IPF models. Gene differential analysis has revealed that MSC-EXOs influence the immune and redox systems in mouse IPF models [69]. Additionally, EXOs derived from human bronchial epithelial cells (HBEC-EXOs) have also been utilized to in the treatment of IPF. Mechanistically, HBEC-EXOs contain miRNAs that inhibit myofibroblast differentiation and cellular senescence. The use of EXOs from healthy lung cells in the treatment of IPF has shown superior efficacy compared to MSC-EXOs, potentially attributable to their miRNA content and lung-homing capabilities [23,70]. EXOs have demonstrated promising therapeutic potential in disease treatment. However, most EXOs secreted without drug loading exhibit limited functionality. 293F cells, which proliferate rapidly in suspension in chemically defined, protein-free, serum-free media, are one of the few well-characterized lines capable of indefinite passaging. A comprehensive characterization of 293F-EXOs has shown that miRNA expression across different EXO batches remains stable. Moreover, these EXOs have shown outstanding preclinical safety in studies conducted both in vitro and in vivo, confirming their potential as effective drug delivery systems [71]. In this study, EXO-siHSP47 was prepared using ultrasonic microfluidics and delivered to activated fibroblasts, where it effectively silenced HSP47 expression. Western blotting and immunofluorescence confirmed that EXO-siHSP47 significantly inhibited HSP47 expression in activated HFL-1 cells and reduced COL-1 secretion, leading to decreased ECM deposition. Wound healing and Transwell migration assays demonstrated that EXO-siHSP47 effectively inhibited fibroblast differentiation and migration.

Although the results are encouraging, it is essential to acknowledge several limitations inherent to the study. Firstly, the inhibitory effect of siHSP47 was exclusively examined in the FMT within fibroblasts. IPF is a complex disease involving the activation of fibroblasts and myofibroblasts induced by dysregulated epithelial, immune, and endothelial cells [72]. Consequently, a more comprehensive assessment that includes additional pathways, such as EMT, is warranted to fully ascertain the silencing efficacy of siHSP47. Secondly, current study’s scope was confirmed the uptake of EXOs by fibroblasts and the effect of EXO-siHSP47 in inhibiting fibroblast overactivation and ECM deposition at the cellular level at the cellular level. In vivo studies using mouse models would be valuable to provide additional safety and efficacy data for clinical trials.

## 5. Conclusions

In conclusion, a novel therapeutic strategy for the treatment of IPF was proposed by utilizing EXOs as drug delivery vehicles. We used an innovative ultrasound microfluidic to efficiently load siHSP47 into EXOs. The results demonstrated that the average loading efficiency of siHSP47 reached 31.1%, surpassing electroporation, incubation and sonication methods. Further studies revealed that EXO-siHSP47 can be successfully penetrated the collagen barrier and delivered into activated fibroblasts and effectively silence the expression of the HSP47. This subsequently led to the improper folding and secretion of collagen into the extracellular space, directly resulting in a reduction of ECM deposition. Moreover, the downregulation of HSP47 expression inhibited the fibroblasts differentiation and migration. These changes are importance for slowing or even reversing the pathological progression of IPF.

This study not only demonstrates the potential of EXO-siHSP47 as a promising antifibrotic therapeutic but also presents a novel EXO-based drug loading platform. It provides significant insights into the utilization of EXOs for the efficient delivery of siRNA, miRNA, peptides, and other therapeutic biomolecules. Looking ahead, further optimization of the EXO delivery system could improve targeting efficiency and therapeutic outcomes, particularly in the fibrotic lung environment. Combining EXO-siHSP47 with other antifibrotic agents may also enhance its efficacy, potentially revolutionizing the treatment of conditions characterized by excessive ECM deposition and tissue fibrosis.

## Data Availability

The authors confirm that the data supporting the findings of this study are available within the article and its Supplementary Materials.

## Supplementary Materials

The following supporting information can be downloaded at: https://www.mdpi.com/article/10.3390/ijms26010382/s1.
