# Supplementary figures and images for: Ultrasonic Microfluidic Method Used for siHSP47 Loaded in Human Embryonic Kidney Cell-Derived Exosomes for Inhibiting TGF-β1 Induced Fibroblast Differentiation and Migration
Source: Int J Mol Sci. 2025 Jan 4;26(1):382. doi: 10.3390/ijms26010382 (PMC11722050; doi:10.3390/ijms26010382)

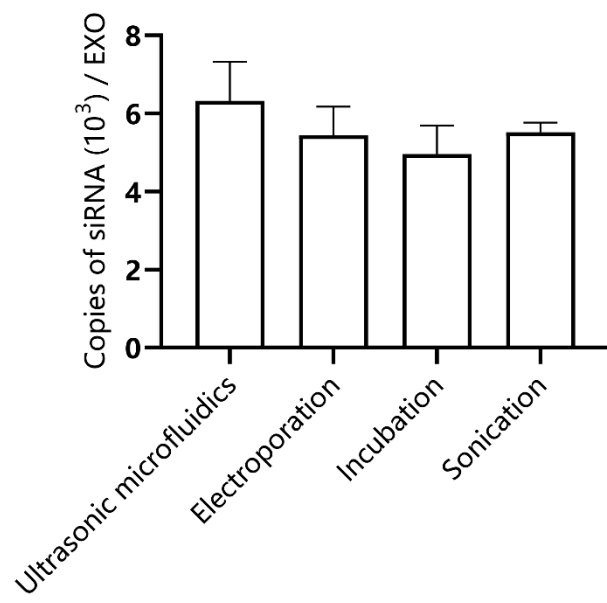

Supplementary Figure S1. The number of siRNA copies loaded into 293F-EXOs by different methods.

Supplement: Supplementary file 1 [file ijms-26-00382-s001.zip › ijms-3346964-supplementary.pdf]
